# Whole-genome 5-hydroxymethylation profiling of human neurons for regulatory and biological insight

**DOI:** 10.1186/s13072-026-00703-z

**Published:** 2026-10-05

**Authors:** Dennis Klose, Mohammad Hossein Sepehri, Remi-André Olsen, Ha Vu, Jason Ernst, Lara Kular, Maria Needhamsen, Maja Jagodic

**Affiliations:** 1 Department of Clinical Neuroscience, Center for Molecular Medicine, Karolinska Institutet, Karolinska University Hospital, 17176 Stockholm, Sweden; 2 Department of Psychiatry, Amsterdam UMC, Vrije Universiteit Amsterdam, Oldenaller 1, 1081HJ Amsterdam, The Netherlands; 3 SciLifeLab, Department of Biochemistry and Biophysics, Stockholm University, Frescativägen 40, 17121 Solna, Sweden; 4 Department of Biological Chemistry, University of California Los Angeles, 615 Charles E Young Dr S, Los Angeles, CA 90095 USA

**Keywords:** Epigenetics, DNA hydroxymethylation, DNA methylation, Neurons, NeuN, Sequencing, Oxford Nanopore Technology

## Abstract

While 5-methylcytosine (5mC) DNA methylation is a well-known player in genome stability and transcriptional regulation, the role of 5-hydroxymethylcytosine (5hmC), particularly prevalent in neurons, remains largely unknown. Here, we used long-read Oxford Nanopore Technology (ONT) to profile whole-genome, native 5mC and 5hmC levels in sorted neuronal nuclei samples from human post-mortem brain tissue. Genomic annotation revealed high 5hmC levels in actively transcribed genes and enhancers, which were enriched in neuron-related pathways, with functional variety when stratifying across chromatin states. Furthermore, 5hmC and 5mC modifications were enriched at transcription factor binding sites, affecting specific downstream regulatory networks. Altogether, our study demonstrates the potential of 5hmC and 5mC DNA modifications to provide biological insight into the regulatory landscape and function of human neurons.

**Supplementary Information:**

The online version contains supplementary material available at https://doi.org/10.1186/s13072-026-00703-z.

## Introduction

5-methylcytosine (5mC) is the most studied epigenetic modification originally described in mammals in 1948 and since then characterized to be involved in regulating gene expression, guarding genome stability and orchestrating development [1, 2]. The process of active oxidation-dependent demethylation mediated by ten-eleven translocation (TET) enzymes was described in 2009 [3]. This process involves the conversion of 5mC to 5-hydroxymethylcytosine (5hmC), 5-formylcytosine (5fC) and 5-carboxylcytosine (5caC), followed by TDG-mediated base excision repair that finally restores the unmodified cytosine [4]. In addition to being an intermediate mark during active demethylation, 5hmC was found to be particularly enriched in neurons implicating its functional role [5–8]. While the regulatory mechanisms are not yet clearly understood, levels of 5hmC in gene bodies, chromatin enhancer states and transcriptional start sites (TSS) have been found to be associated with gene expression in a tissue-specific manner [3, 4, 9].

Traditionally, 5mC levels have been measured by methods such as whole genome bisulfite sequencing (WGBS), reduced representation bisulfite sequencing (RRBS) or Illumina BeadChips DNA methylation arrays (27K, 450K, 850K) following treatment with sodium bisulfite (BS) [10]. While broadly used and generally reliable, those methods encompass different biases and shortcomings, such as (i) degradation of DNA, due to the abrasive chemical treatment of the genomic DNA with BS for conversion of non-methylated cytosines, (ii) complexity reduction of the genome to three nucleotides, and (iii) inability to cover a large representative amount of CpG positions throughout the entire genome [11]. Importantly, BS conversion does not enable discrimination between true 5mC and 5hmC, resulting in all these methods quantifying a net effect of the two modifications. The detection of 5hmC typically requires another parallel treatment of a separate aliquot either with an oxidative chemical reagent (oxBS) or an enzymatic reaction referred to as Tet-assisted bisulfite (TAB) [12]. 5hmC levels are subsequently calculated indirectly, for example through subtraction of oxidative BS (true 5mC) signals from total BS [13]. Thus, the accurate estimation of true 5mC and 5hmC modifications remains insufficiently resolved.

The possibility to detect native 5mC and 5hmC modifications simultaneously from the same sample on a genome-wide level with Oxford Nanopore Technology (ONT) opens up a new approach to epigenetic analyses [14]. ONT sequencing omits BS and enzymatic conversion steps and profiles native genomic DNA without potential amplification bias as commonly introduced in traditional methods. Rather, nucleotide modifications are detected as changes in electric currents when long, native genomic DNA fragments pass through pores [15]. Base- and modification calling algorithms, trained on existing genetic and epigenetic data using deep learning, were developed together with the technology [16].

In this study, we sought to explore the biological insights that can be obtained from genome-wide native 5hmC profiling in human neurons using ONT sequencing and to evaluate its utility for characterizing the transcriptional regulatory genomic landscapes.

## Methods

### Sample preparation and sequencing

NeuN^+^ nuclei were isolated from post-mortem brain specimen using fluorescence activated nuclei sorting (FANS) as previously described [17]. Fragmented genomic DNA was added to the *Genomic DNA by Ligation (SQK-LSK109)* Nanopore library preparation protocol following manufactory’s guidelines. Subsequently, libraries were sequenced on the R9.4.1 PromethION flow cell (FLO-PRO002) with the following run settings: −165 mV Initial Bias Voltage and 90 min Mux scan period.

### Base and DNA methylation calling

Base calling was conducted using Guppy v.5.0.16 with the default high-accuracy (HAC) model through Megalodon v.2.5.0. DNA methylation (5mC and 5hmC) calling in CpG context was conducted using the Remora (https://github.com/nanoporetech/remora) HAC model: dna_r9.4.1_e8 hac 0.0.0 5hmc_5mc CG, whereas DNA methylation calling of 5mC in all contexts, referred to as CpX, was conducted using the following config file: res_dna_r941_prom_modbases_5mC_v001.cfg. Subsequently, .FASTQ files were aligned to the GCA_000001405.29_GRCh38.p14 human reference genome (NCBI) with minimap2 v.2.24 [18]. A schematic pipeline overview, read length and coverage assessment are shown in Supp Fig. 1. A conversion table (https://genome.ucsc.edu/cgi-bin/hgTracks?db=hg38&chromInfoPage=) was used to convert alias chromosome names to “chr1”, “chr2” etc. and the coverage2cytosine function from Bismark v.0.24.2 [19] to distinguish CpG and CpH (= CHG and CHH) sites. Raw (fast5) sequencing and the processed data are available upon reasonable request .

**Fig. 1:**
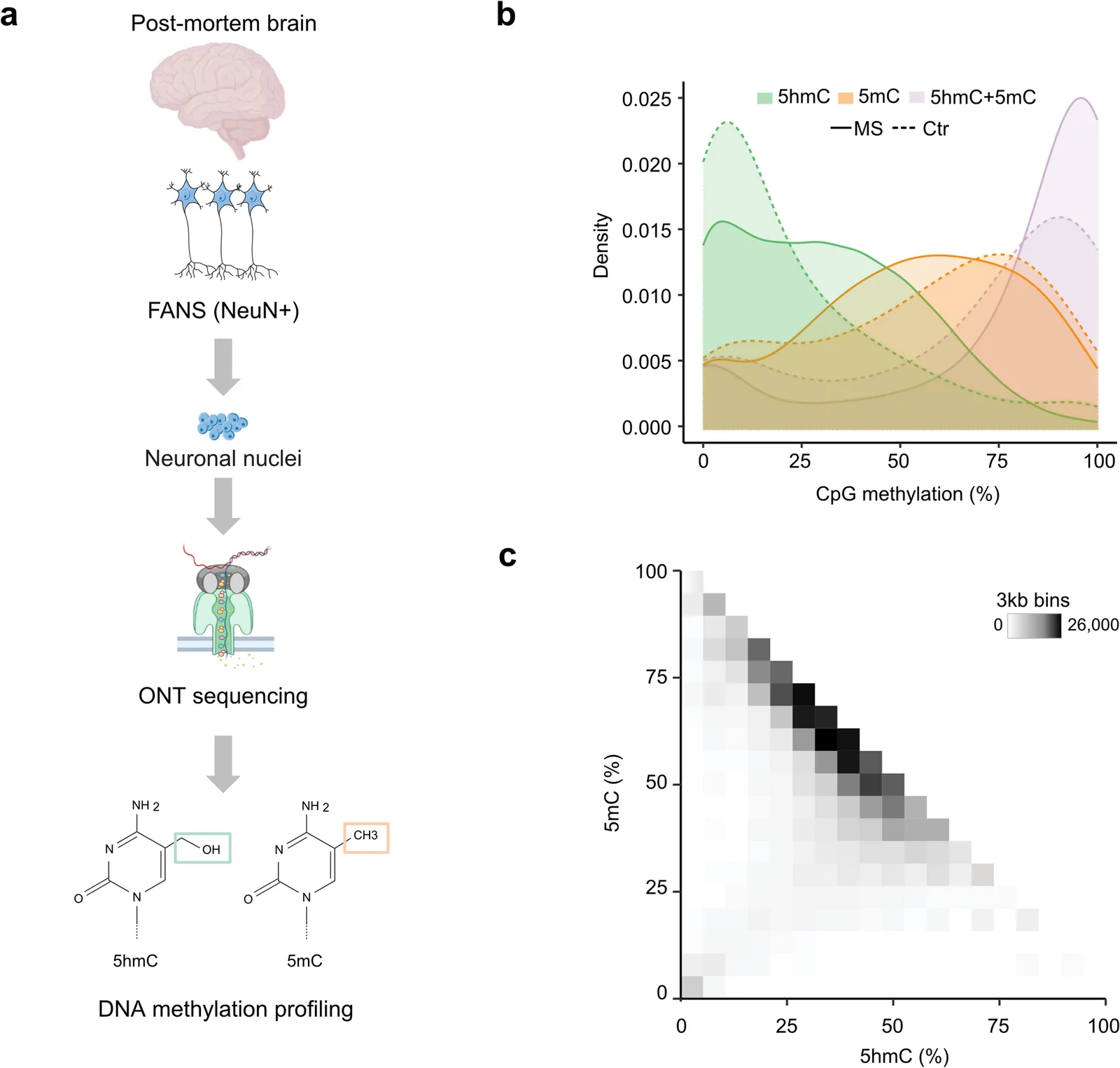
Study design and Oxford Nanopore Technology sequencing. **a** Isolated nuclei were sorted from postmortem brain samples based on NeuN-staining intensity. Genomic DNA was extracted from sorted NeuN+ nuclei, fragmented and profiled with Oxford Nanopore Technology (ONT). **b** Density distribution of 5hmC (green), 5mC (orange), and total 5hmC+5mC (purple) DNA methylation levels (%) in CpG context of MS (solid line) and control (dashed line) samples. **c** Comparison of 5mC and 5hmC levels (%) in 3 kb bins. The white-to-black color gradient illustrates the overlap density

### Annotation of 5hmC and 5mC marks

Reads with less than either 5X or 10X coverage and those mapping to the X- and Y-chromosomes were excluded. DNA methylation levels reported per cytosine were quantified as methylated C/total coverage in R, except for gene body methylation levels, where the command line program CpGtools was used [20]. Estimation of mean 5mC and 5hmC across genomic and regulatory features was conducted with SeqMonk v1.48.1 in 3 kb running bins, using the UCSC annotation (downloaded 20th May 2020). The summary 18-state ChromHMM from healthy brain samples, were obtained from the International Human Epigenome Consortium (IHEC) [21]. Closest genes within ±2 kb of a chromatin state region (incl. overlapping) were determined using the “Annotate probe report” tool from SeqMonk v1.48.1.

### Ingenuity pathway analysis

The gencode.v38.annotation.gtf file and summary 18-state ChromHMM annotations from healthy brain samples from the IHEC Consortium portal () were used to annotate genes and chromatin states for Ingenuity Pathways Analysis (IPA) [22]. Promoters were defined as regions 1.5 kb upstream of the TSS and exon 1, that overlapped with chromatin states 1_TssA, 2_TssFlnk, 3_TssFlnkU, 4_TssFlnkD, 14_TssBiv or 15_EnhBiv. Transcribed regions (Tx) were defined as exons overlapping with chromatin states 5_Tx and 6_ TxWk. Enhancers were defined as regions overlappiing with chromatin states 8_EnhG2, 9_EnhA1, 10_EnhA2 and 11_EnhWk. Repressed polycomb (PC) were defined as regions overlapping with chromatin states 16_ReprPC or 17_ReprPCWk. Zinc finger proteins (ZNF) were defined as regions overlapping with chromatin state 12_ZNF/Rpts regions, subsequently filtered for ZNF genes. Sites with 10X coverage and 5hmC > 60% were considered for IPA.

### Transcription factor motif enrichment analysis

The CentriMo tool [23], available through the MEME suite [24], was utilized for transcription factor (TF) motif enrichment analysis. CpGs passing the 10X coverage threshold with 5hmC > 60% or 5mC > 80% were selected and extended 15 bp up- and down-stream of their target CpG. For the relative enrichment analysis, CpGs with 0% DNA methylation were utilized as the background to identify motifs preferentially enriched in highly modified regions. In addition, an absolute enrichment analysis implemented in CentriMo was performed to assess the robustness of the results to background selection. The JASPAR core (2024) vertebrates, non-redundant DNA motif database was selected [25]. Result output files were filtered for CpG consensus, and TFs with adj.Pval < 0.01 were considered significant.

### Transcriptional regulatory network analysis

Protein-coding target genes of significantly enriched transcription factors were predicted using JASPAR 2024 v0.99.7 motifs, based on position weight matrices (PWM) [25]. A *± 15* bp window around each CpG was scanned using TFBSTools v.1.46.0 [26] with BSgenome.Hsapiens.UCSC.hg38 as reference v.1.4.5 [27]. Target gene expression was inferred from gene body 5hmC levels (excluding exon 1), with a threshold of >60% defined as active transcription. Transcriptional regulatory networks were rendered with visNetwork (v2.1.4) [28] and visualized in CytoScape [29] with TFs as nodes and edge colors representing enriched or depleted TF target genes estimated with Fisher’s exact test. Functional interpretation was assessed with GO enrichment (clusterProfiler v.4.16.0 [30]) and per-TF enrichment bobble plots were generated with ggplot2 v.4.0.0. Integration with the Human Protein Atlas single-nuclei brain data [31] allowed visualization of TF and target gene expression levels across brain cell types. Different parameters, including PWM, window size and 5hmC thresholds were assessed using the 5hmC_regulator shinyApp, which, apart from JASPAR 2024 v.0.99.7, included several additional resources such as DoRothEA regulons v.1.20.0 [32], CoLlecTRI via decoupleR v.2.140 [33, 34], TRRUST v.2 [35], and allows for upload of user-supplied TF-target tables. For interpretation GO, Reactome (clusterProfiler v.4.16.0 [30] and ReactomePA v.1.52.0 [36]) were included. Access to the 5hmC_regulator shinyApp can be found here: https://tf-regulatory-network-builder.jagodiclab.com/.

### Statistical analysis

Statistical analyses were performed in RStudio v.2023.06.1+524 using R language v.4.3.1, unless stated otherwise. For plotting, we used the ggplot v.3.4.3, ggpubr v.0.6.0, ggVennDiagram v.1.5.4 [37] and ComplexHeatmap v.2.24.1 [38] packages, for general data manipulations we used dplyr v.1.1.3, tidyr v.1.3.0 and stringr v.1.5.0 [39]. For statistics we used the base R functions cor.test, lm and poly, such as rstatix v.0.7.2 functions wilcox_test and wilcox_effsize. For creating heatmaps we used the pheatmap package v.1.0.12 with the complete linkage clustering method.

## Results

### 5mC and 5hmC methylome profiling of human brain neurons

For genome-wide profiling of 5hmC and 5mC levels (0%-100%), we applied Oxford Nanopore technology (ONT) to neuronal DNA isolated from NeuN^+^ nuclei sorted from post-mortem brain tissues from a person with (pw)MS and a non-neurological control (Ctr liver and lung cancer) (Fig. 1a, Supp Table 1). DNA methylation density plots confirmed expected bimodal distributions in combined 5hmC+5mC levels, as well as distinct 5hmC and 5mC signals. Noticeably, while the total (5hmC+5mC) levels were similar, the 5hmC and corresponding 5mC levels differed between MS and Ctr samples. Both peaked at 0% 5hmC, however, the peak was higher in Ctr, while MS displayed a larger shoulder indicating intermediate 5hmC levels (Fig. 1b), also reflected in the median 5hmC levels of 30% and 13.1% for MS and Ctr samples, respectively.

Comparison of mean 5hmC and 5mC levels in 3 kb windows, which is based on the same number of CpGs and sequencing coverage, revealed that 25–50% 5hmC levels most often coincide with 50–75% 5mC levels (Fig. 1c). High 5mC (80–100%) and low 5hmC (0–10%) were also found, while the opposite scenario of high 5hmC and low 5mC was less common.

To technically confirm our results, we compared ONT-derived DNA methylation levels, filtered for 5X or 10X coverage, against BS/oxBS-based Illumina BeadChip 450 K technology applied to the same samples, which showed overall high consistency across 5mC and 5hmC levels, although agreement decreased at CpGs with high estimated 5hmC levels (Supp. Fig. 2).

Taken together, these results suggest that separating the 5hmC and 5mC signal in neurons can provide complementary and additional epigenetic insight.

### Distribution of neuronal 5hmC and 5mC across genomic features

**Fig. 2:**
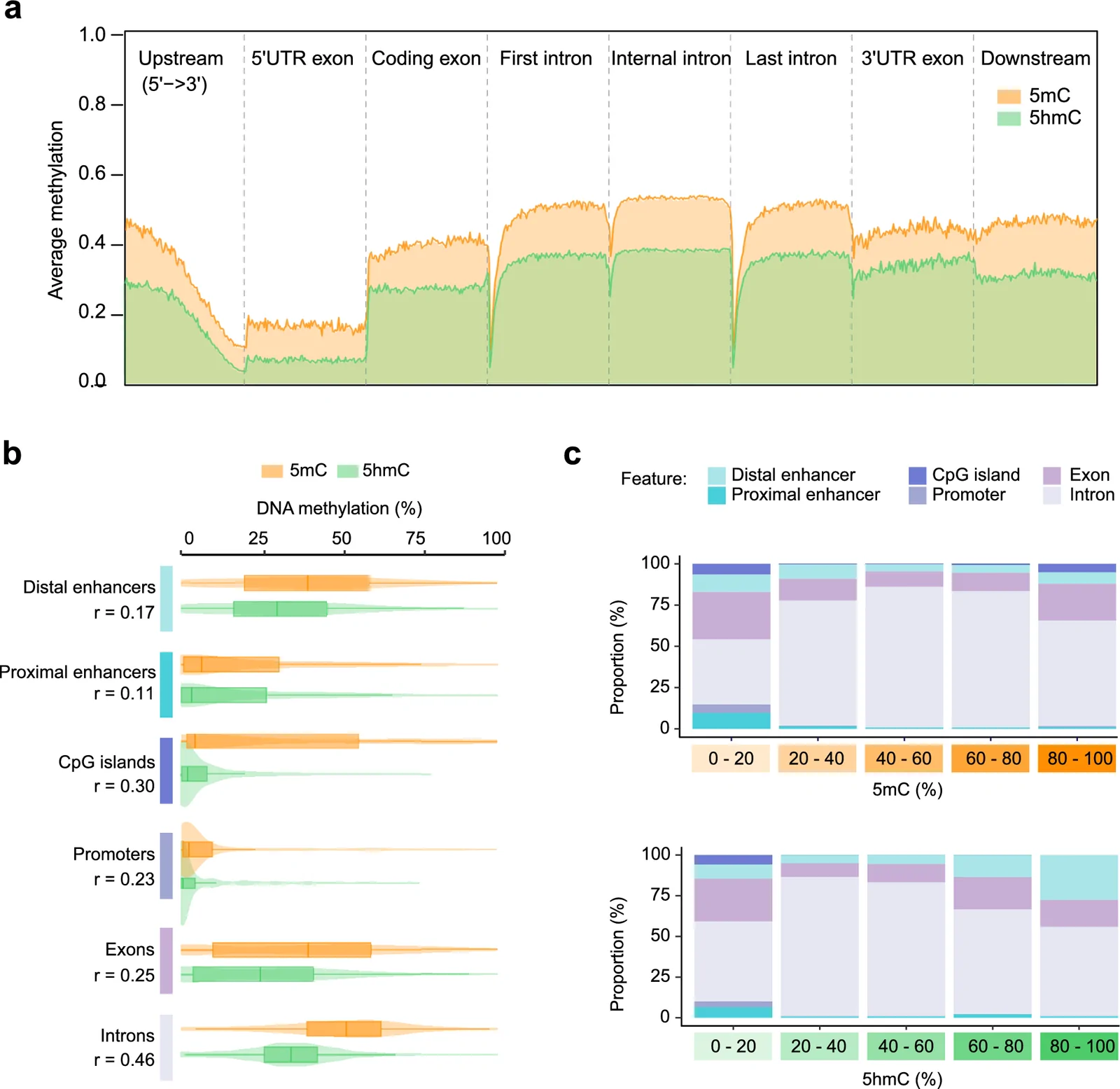
Genomic feature annotation of neuronal 5hmC and 5mC levels. DNA methylation distributions of 5hmC (green) and 5mC (orange) levels (%) accessed across different **a** gene- and **b** regulatory features. UTR: Untranslated region, r: Wilcox effect size. **c** Stacked bar plots illustrating the proportion of different features in segregated 20% bins of 5mC (top) and 5hmC (bottom) DNA methylation levels

To explore the genomic distribution of 5hmC compared to 5mC, we examined their average levels across different gene body features. 5hmC levels were generally lower than 5mC levels but followed a similar pattern across the gene (Fig. 2a). Intronic CpGs displayed the highest 5mC (50%) and 5hmC (40%) levels, followed by 3’UTR, downstream and coding exon sequences, whereas promoter-like (i.e., upstream and 5’UTR) features exhibited the lowest 5mC (10–45%) and 5hmC (5–30%) levels (Fig. 2a). Noticeably, DNA methylation levels of both 5hmC and 5mC decreased at exon-intron junctions (Fig. 2a).

We further explored whether and to what extent 5mC and 5hmC levels differ between generically identified regulatory features such as promoters, proximal enhancers, distal enhancers and CpG islands (CGIs). Comparison of the methylation levels within each feature identified the largest Wilcox effect size differences (indicated with r) between 5mC and 5hmC in introns, with median levels of 52% and 34%, respectively (Fig. 2b). While the distribution of features was similar across low to intermediate 5mC and 5hmC levels, differences emerged at high methylation ranges (Fig. 2c). Indeed, the proportion of distal enhancer elements increased in regions with higher levels of 5hmC, whereas their proportions remained stable across 5mC levels. However, this observation should be carefully interpreted given the few sites with high 5hmC levels as shown in Fig. 1b and c and median distal enhancer 5hmC levels around 30% (Fig. 2b). On the contrary, the proportion of CGIs decreased in regions with higher 5hmC levels, whereas CGIs persisted at the high end of the 5mC methylation range (80–100%) (Fig. 2c).

Thus, while 5hmC and 5mC levels differ, their distribution across regulatory features appears similar at low to intermediate methylation levels, whereas differences are observed in introns, distal enhancers and CGIs in the regions with high methylation levels.

### 5hmC levels are elevated in enhancers and transcribed genes of the human brain

**Fig. 3:**
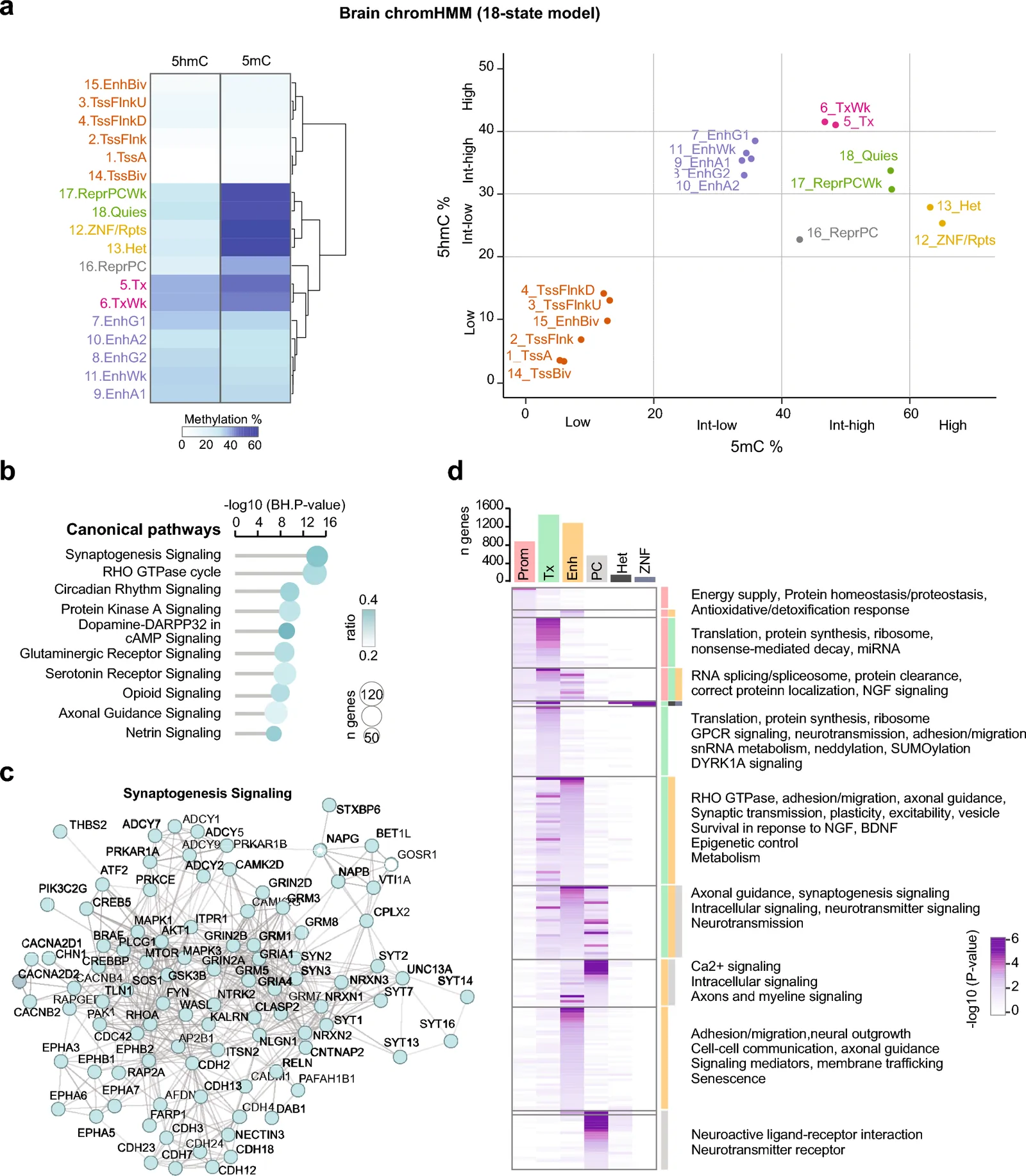
5hmC and 5mC represent different brain-derived chromatin states and have functional relevance for neurons. **a** Heatmap illustrating average 5hmC and 5mC levels (%) within the 18-state healthy brain summary ChromHMM models. DNA modifications levels are depicted in a white-to-blue colour gradient. Scatterplot of average 5hmC (%) and 5mC (%) levels within brain summary ChromHMM states color-coded according to their classification (low-intermediate-high). TssA: Active Transcriptional start sites (TSS), TssFlnk: Flanking TSS, TssFlnkU: Flanking TSS upstream, TssFlnkD: Flanking TSS downstream, Tx: Strong transcription, TxWk: Weak transcription, EnhG1: Genic enhancer 1, Enh2: Genic enhancer 2, EnhA1: Active enhancer 1, EnhA2: Active enhancer 2, EnhWk: Weak enhancer, ZNF/Rpts: Zinc finger protein genes and repeats, Het: Heterochromatin, TssBiv: Bivalents/poised TSS, EnhBiv: Bivalent enhancer, ReprPC: Repressed Polycomb, ReprPCWk: Weak repressed Polycomb, Quies: Quiescent/low. **b** Significant canonical pathways predicted from Ingenuity Pathway Analysis (IPA) based on high-5hmC (> 60%) input genes. The significance level (-log10(BH-Pval), BH: Benjamini–Hochberg) is depicted in a white-to-turquoise color gradient. The circle size reflects the number of genes (n) represented in each pathway. **c** Gene network illustration of the top-enriched Synaptogenesis Signaling pathway. **d** Heatmap illustrating enriched canonical pathways based on high-5hmC (> 60%) genes segregated into: Prom: Promoters; Tx: Transcribed; Enh: Enhancers; PC: Polycomb; Het: Heterochromatin and ZNF: ZNF genes. The significance level (-log10(Pval)) is depicted in a white-to-purple color gradient

For additional regulatory insight, we next exploited chromatin states summarized across “healthy brain” samples from IHEC. Chromatin states separated according to their 5hmC and 5mC levels (Fig. 3a) and stratification into (1) low (0–20%), (2) intermediate(I)-low (5hmC: 20–30%, 5mC: 20–40%), (3) intermediate(I)-high (5hmC: 30–40%, 5mC: 40–60%), and (4) high (5hmC: 40–100%, 5mC: 60–100%), further enhanced the separation (Fig. 3a). “Low 5hmC & low 5mC” classification represented predominantly states associated with promoter (or TSS) states as well as bivalent enhancers (EnhBiv), “I-high 5hmC & I-low 5mC” represented enhancer states, “high 5hmC & I-high 5mC” included states associated with active transcription (Tx) states. “I-high 5hmC & I-high 5mC” encompassed states representing quiescent/weak polycomb (Quies and ReprPCWk) repressed regions, whereas states repressed by polycomb (ReprPC) classified separately based on “I-low 5hmC & I-high 5mC” levels. Finally, repressed heterochromatin (Het) and zinc finger (ZNF) repeats (Rpts) states associated with “I-low 5hmC & high 5mC” (Fig. 3a). Hence, combinatorial patterns of 5hmC and 5mC distinguish brain-derived chromatin states in human neurons, highlighting their potential to give insight into neuronal chromatin architecture and transcriptional activity [40].

To gain biological insight into the relevance of 5hmC, we performed pathway analysis using Ingenuity Pathways analysis (IPA) of all genes with high (> 60%) 5hmC levels within 2 kb proximity (n = 3838 genes). Results showed enrichment of canonical pathways associated with neuronal functions, particularly synapse formation, axonal guidance and neurotransmitter signaling, along with general intracellular pathways related to Rho GTPase, protein kinase A and circadian rhythm signaling (Fig. 3b, Supp. Table 1). The genes implicated in the top term “Synaptogenesis signaling” encode several adhesion molecules of the ephrin, cadherin, neuroligin, neurexin, reelin, synaptotagmin, synapsin, and SNAP families together with multiple subunits of glutamate receptors, calcium channels and Rho GTPases, MAPK, cAMP, CREB and downstream signaling (Fig. 3c, Supp. Table 1). To further refine the functional interpretation of 5hmC, genomic regions were next annotated according to brain-specific chromatin states, which were grouped into broader functional categories based on shared regulatory properties and similar 5hmC/5mC profiles. This complementary analysis implied biological relevance of high 5hmC levels in transcribed (Tx) regions, enhancers and PC repressed segments (Fig. 3d, Supp. Table 1). More specifically, enriched pathways overlapping with Tx genes were related to common cellular functions such as protein synthesis and splicing, as well as neuron-related functions such as neurotransmission and axonal guidance. Pathways enriched in genes overlapping with enhancers and Polycomb-repressed chromatin states seemed neuron-specific and included terms such as Ca^2+^ signaling, axons and myelin signaling, neural outgrowth and neuroactive ligand-receptor interaction (Fig. 3d, Supp. Table 1).

Altogether, our data imply that genome-wide profiling of 5hmC and 5mC can provide biologically meaningful insight into neuronal regulatory mechanisms, with chromatin state–specific patterns revealing refined functional associations relevant for neuronal identity and activity.

### Neuronal 5hmC denotes transcriptional regulation

**Fig. 4:**
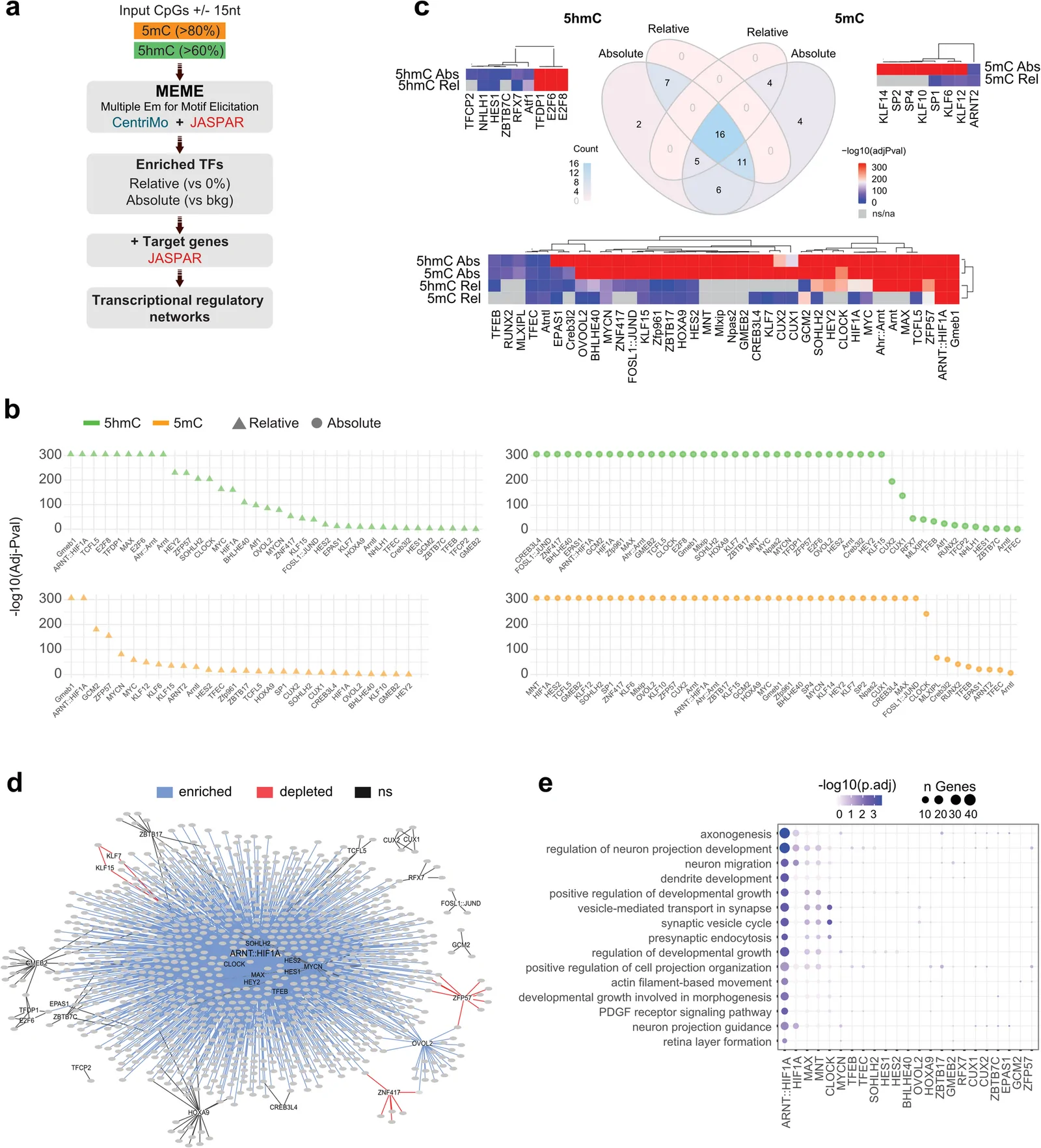
5hmC and 5mC denote transcriptional regulation. **a** Flowchart illustrating strategy for transcription factor (TF) enrichment and subsequent regulatory network analyses. Bkg: background. **b** Scatterplots illustrating significantly enriched TFs (x-axis) ranked according to -log10(adj.Pval). TFs enriched in high-5hmC (green) and high-5mC (orange) sites, above 60% and 80%, respectively, either in comparison to 0% DNA methylated sites (relative, triangle) or against the background (bkg) (absolute, circle) were plotted. **c** Venn diagram showing overlap between significantly enriched TFs in high-5hmC (60%) and high-5mC (80%) based on absolute or relative approaches, respectively. The color key (blue-to-pink) illustrates the overlap (high-to-low) of enriched TFs. Heatmaps for high-5hmC (left), high-5mC (right) or overlapping (bottom) TFs. The color key (red-to-blue) illustrates the -log10(adjPval) of the relative (rel) or absolute (abs) enrichment of significantly enriched TFs (high-to-low). **d** Network of high-5hmC TFs (nodes) and their predicted target genes with high-5hmC (grey circles). Edges colors illustrate whether target genes are significantly enriched (blue), depleted (red) or not significant (black) with Fisher’s exact test. **e** Bobble plot of top-15 GO pathways (rows) with bobble sizes illustrating number of target genes for different TFs (columns). The color key (blue-to-white) illustrates -log10(adjPval)

Next, we sought to further explore the regulatory potential of high 5hmC (> 60%) and 5mC (> 80%) regions by conducting transcription factor (TF) motifs enrichment analysis (Fig. 4a). A total of 34 TFs were significantly enriched (adj.Pval < 0.01), when compared to 0% 5hmC (relative analysis, Fig. 4b) with highly significant TFs including Gmeb1, ARNT::HIF1A, TCFL5, E2F8, TFDP1, MAX, E2F6, Ahr::Arnt and Arnt. Noticeably, Gmeb1 and ARNT::HIF1A were also highly enriched in high (> 80%) vs. low (0%) 5mC relative analysis. An additional 13 and 21 TFs passed the adj.Pval < 0.01 threshold for 5hmC and 5mC, respectively, when the absolute enrichment against the genomic background was considered (absolute analysis, Fig. 4b). While nearly 70% of TFs (38 of 55) were significant in regions with both high 5hmC and 5mC, several were specifically associated with either 5hmC, such as E2F8, E2F6 and TFDP1, or 5mC, such as SP1, SP2, SP4 and KLF6, KLF10, KLF12, KLF14 (Fig. 4c). Considering both the relative and absolute analyses for 5hmC and 5mC, the top enriched TFs were Gmeb1, ARNT::HIF1A and ZFP57 (Fig. 4c).

Given that not only enhancers, but also transcribed genes overlap with 5hmC (Fig. 3d), we next set out to explore transcriptional regulatory networks based on 5hmC-enriched TFs and their 5hmC-high target genes. A dense network formed around ARNT::HIF1A, CLOCK, MAX, and MNT, amongst other TFs (Fig. 4d). GO analysis of network genes showed enriched pathways representing predominantly neuronal and neurodevelopmental related processes, such as axonogenesis, regulation of neuron projection development and neuron migration, with per-TF enrichment confirming the involvement of previously mentioned TFs, especially ARNT::HIF1A (Fig. 4e). Noticeably, neither Gmeb1 nor ZFP57 was located in the dense part of the network, despite being identified as top-enriched TFs. This could be explained by the absence of Gmeb1 in the JASPAR human motif database, and that only 9 (of 98) predicted ZFP57 target genes exhibited high 5hmC levels. Other significantly depleted genes included targets of ZNF417, KLF15 and KLF7. Noticeably, according to the single nuclei data from the Human Protein Atlas [31], ZFP57 and KLF15 are predominantly expressed in oligodendrocytes and astrocytes, respectively (supp. Figure 3), supporting the likelihood that their target genes are not highly expressed in neurons. Importantly, these observations also suggest that our approach is capable of capturing not only active but also repressed regulatory programs in a neuron-specific manner, thereby adding an extra layer of insight when exploring transcriptional regulatory networks based on 5hmC-enriched TFs and their targets genes.

## Discussion

In this study, we profiled the whole-genome distribution of 5mC and 5hmC in sorted human neurons using ONT sequencing and compared against array-based BS/oxBS method applied to the same samples. We characterized 5mC and 5hmC across genomic and regulatory features in brain-derived chromatin states. Furthermore, we conducted pathway analysis and inferred transcriptional regulatory networks based on high 5hmC regions.

We found that the ONT-based 5mC and 5hmC detection is similar to previously BS/oxBS array-derived quantification [17], even though discrepancies appeared in a base-to-base comparison especially for high 5hmC levels. Notably, this comparison was based on 171 999 and 14 750 CpG sites after applying 5X or 10X coverage filter, respectively, with the vast majority of sites falling into the low-5hmC level category. Therefore, this orthogonal comparison should be regarded as technical support for the overall genome-wide methylation profiles, rather than locus-specific validation. Furthermore, global 5mC and 5hmC levels matched those reported in previous studies investigating neuronal 5hmC and 5mC levels, including genome-wide profiling by TAB-seq in mouse frontal cortex [41] and BS/oxBS-sequencing of sorted mouse neurons [42]. While the genome-wide 5hmC distribution compared between the MS and control sample was consistent with previous reports of elevated neuronal 5hmC in MS [17], the comparison was intended only to illustrate the potential of ONT sequencing to detect differences in genome-wide 5hmC distributions. Accordingly, no conclusions regarding disease-associated epigenetic alterations can be drawn from this comparison.

Our detailed characterization of 5mC and 5hmC across the gene body segments indicated similar distributions between 5mC and 5hmC. Yet, this should be regarded as an average for all genes in neurons and further stratification according to neuronal vs. non-neuronal genes, or different neuronal subtypes, might unveil differences, notably in 5hmC levels, as observed in a study using immunoprecipitation in mouse neurons [6]. Another study exploiting reduced representation oxBS sequencing to investigate 5mC levels in CpG-dense regions in human brain samples concord with our findings on 5mC levels in promoters, exons and introns [43]. Further, we observed that 5hmC is largely absent from CpG islands (CGIs) and therefore differs from 5mC across this regulatory feature. Low 5hmC in certain CGIs can be partially supported by results from immunoprecipitation experiments in mouse neuronal progenitor cells, showing that 5hmC is also absent from p300 regions, a CpG-dense regulatory feature [44].

Chromatin state predictions from epigenetic marks with machine learning-based algorithms, such as ChromHMM [45], is a well-established method for assigning tissue-specific regulatory insight. Here we explored a recent summary annotation across healthy brain samples from IHEC and found refined grouping of functionally related chromatin states based on 5mC and 5hmC levels. Promoter-associated states exhibited low 5mC levels, whereas heterochromatin showed high 5mC levels, consistent with established epigenetic principles. Furthermore, relatively high 5hmC levels were identified across enhancers and states associated with active transcription in the brain tissue, consistent with previous findings [46], further supporting the association between 5hmC enrichment and transcriptionally active regulatory regions in neurons.

In a study using AbaSI sequencing, the authors explored 5hmC levels in enhancers and reported elevated 5hmC in poised compared to active enhancers [47]. Notably, their findings are based on histone marks from an adult brain midfrontal lobe sample, whereas we used the summarized chromatin state strategy across multiple IHEC brain samples. Nevertheless, the outcome of pathway-enrichment analysis of 5hmC-enriched genes corroborated the enrichment of terms pertaining to synaptic transmission and neural morphogenesis found by another study using forebrain organoids [48]. While we show that the combination of 5hmC together with 5mC seems to define brain-related chromatin states, it is important to note that, due to coverage limitations, we did not explore strand-specific DNA methylation levels.

Transcription factor enrichment analysis of highly methylated motifs revealed a substantial overlap in significant TFs between 5hmC and 5mC. Notably, amongst top-enriched TFs, ZFP57 and HIF1A have previously been shown to bind in a DNA methylation-sensitive manner [49–51]. Some of the enriched TF motifs appeared to be specific to 5hmC, including E2F8, E2F6, and their TFDP1 dimerization partner, which are known to repress the cell cycle in post-mitotic cells, such as neurons, through polycomb interaction [52–54], as an important survival mechanism in response to stress and DNA damage [55–58]. In contrast, other distinct classes of TFs, including members of the KLF and SP families, were specifically enriched in 5mC motifs. This is particularly interesting in light of a study suggesting that KLF4 exhibits differential affinity for cytosine modifications, potentially allowing a refined response beyond a binary “on/off” scenario for transcriptional regulation of target genes [59]. Interestingly, both pathway enrichment and transcription factor motif analyses highlighted circadian regulation, including enrichment of CLOCK motifs. Together with the enrichment ofARNT ::HIF1A motifs, this raises the possibility that some of the identified regulatory networks may represent dynamic transcriptional programs whose activity varies over time. Because the present study was based on bulk NeuN⁺ nuclei collected at a single post-mortem time point, such temporal variation could not be assessed. Future studies will be required to determine whether 5hmC- and 5mC-associated regulatory networks exhibit temporal dynamics.

Several limitations of the present study should be acknowledged. First, the analyses were based on only two post-mortem human brain samples originating from different disease categories, limiting the ability to distinguish disease-related effects from inter-individual variability. Furthermore, the observed enrichment of DNA methylation within chromatin states, transcription factor binding sites, and biological pathways should not be interpreted as direct evidence of regulatory mechanisms. Validation of these findings will require larger, disease-matched cohorts together with complementary functional studies. Nevertheless, the genome-wide nature of the approach enabled identification of biologically relevant regulatory patterns and pathways in human neurons, providing a valuable framework for future studies investigating the role of 5hmC and 5mC in neurological disease. In addition, the study was performed using the now-decommissioned ONT R9 flow cell chemistry, together with the high-accuracy (HAC) basecalling workflow available at the time of analysis. Since then, newer sequencing chemistries, super-accuracy (SUP) basecalling models, and improved modified-base calling algorithms, including modification probability estimates, have become available and are expected to further improve the accuracy and confidence of 5mC and 5hmC detection. Future studies using newer flow cells with improved modification-calling accuracy in the rapidly evolving field of long-read sequencing may further refine the regulatory insights obtained here.

Altogether, our study highlights the potential for 5mC and 5hmC profiling to provide biological insights into transcriptional regulatory mechanisms shaping neuronal cell identity and function, offering a valuable framework for gaining mechanistic insight into brain development and neurodegenerative diseases.

## Supplementary Information

Supplementary Material 1. Figure S1. Analysis pipeline overview and quality assessment. a) Pipeline overview of Oxford Nanopore Technology (ONT) data preprocessing. Squiggle signals generated by DNA passing through the nanopore membrane protein were transformed into base calls by Guppy. Remora was used for calling epigenetically modified bases, 5hmC and 5mC, in CpGs (cytosine followed by guanine), whereas a combined signal of the two modifications was called in all contexts (CpX). After base and DNA methylation calling, reads passing quality control were mapped to human genome hg38 using minimap2. b) The number of reads (#), read length (kb) and coverage (X) were assessed and both samples reached near whole-genome 1X coverage of 57.7M and 48.9M sites in CpG context for S1 and S2, respectively. Thus, we observed an expected long-read distribution covering the vast majority of CpG sites within the human genome. c) Investigation of 5mC+5hmC levels in a non-CpG context estimated by the “CpX” (5mC+5hmC) model revealed that the majority of non-CpG sites were unmethylated. However, a small proportion of cytosines exhibited DNA methylation levels of ~10%, as indicated by a peak in the density plot. d) Comparison of methylation levels called by the “CpX” (5mC+5hmC) model with the 5mC+5hmC levels called by the “CpG” model had a correlation coefficient (rho) of 0.69 and showed an expected bimodal distribution, with fewer sites falling into the low, compared with the high, DNA methylation category. The color gradient (white-blue-yellow) represents the estimated 2D probability density.

Supplementary Material 2. Figure S2. Comparison of ONT and array-based oxBS/BS technology. Comparison of 5mC (upper panels) and 5hmC (lower panels) levels obtained with ONT as a) 5X coverage, segregated into 10% intervals or b) 10X coverage and compared with site-specific Illumina array-based 450K oxBS/BS methodology. Rho (ρ) represents the Spearman’s correlation coefficient, whilst the color key (blue-to-yellow) illustrates the number of sites compared.

Supplementary Material 3. Figure S3. Expression levels of 5hmC-enriched transcription factors. Heatmap of 5hmC-high enriched transcription factors (columns) across different tissues (rows) available through the human protein atlas. The color key (red-to-white) illustrates expression levels (high-to-low) in count per million (cpm).

Supplementary Material 4. Table S1. Sample Description

## Data Availability

5mC and 5hmC values had previously been obtained from the same two samples, which had been processed in parallel with bisulfite (BS)- and oxidative BS-based methods followed by Illumina Infinium HumanMethylation450KBeadChip (450 K) profiling [17] with data available at the Gene Expression Omnibus (GEO) database under GSE119532. Newly generated ONT data can be accessed upon request.
