# Supplementary material for: Whole-genome 5-hydroxymethylation profiling of human neurons for regulatory and biological insight
Source: Epigenetics Chromatin. 2026 Oct 5;19:41. doi: 10.1186/s13072-026-00703-z (PMC13644222; doi:10.1186/s13072-026-00703-z)

Supplementary Figure 2

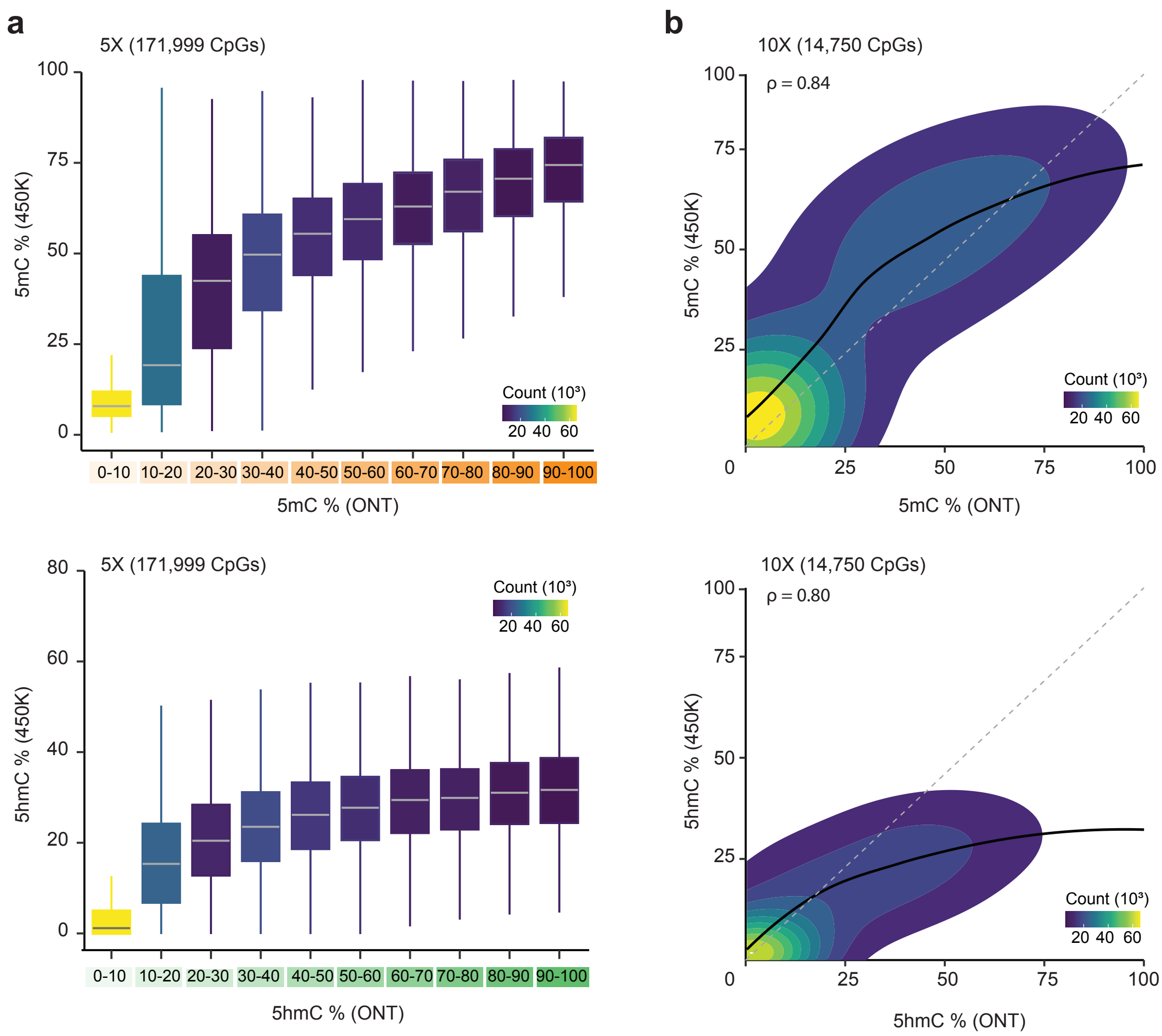

Supplement: Supplementary file 2 [file 13072_2026_703_MOESM2_ESM.pdf]
